# Thyroid Carcinoma: Phenotypic Features, Underlying Biology and Potential Relevance for Targeting Therapy

**DOI:** 10.3390/ijms22041950

**Published:** 2021-02-16

**Authors:** Jinwei Hu, Isabella J. Yuan, Saied Mirshahidi, Alfred Simental, Steve C. Lee, Xiangpeng Yuan

**Affiliations:** 1 Department of Otolaryngology-Head and Neck Surgery, Loma Linda University Medical Center, Loma Linda, CA 92354, USA; ouyangsnow99@gmail.com (J.H.); izzyyuan99@gmail.com (I.J.Y.); asimental@llu.edu (A.S.); stevec.lee4@va.gov (S.C.L.); 2 Cancer Center Biospecimen Laboratory, Loma Linda University Medical Center, Loma Linda, CA 92354, USA; mirshahidis@llu.edu

**Keywords:** thyroid carcinoma, heterogeneity, cancer stem cells, tumor microenvironments, genetics, epigenetics, targeting therapy

## Abstract

Thyroid carcinoma consists a group of phenotypically heterogeneous cancers. Recent advances in biological technologies have been advancing the delineation of genetic, epigenetic, and non-genetic factors that contribute to the heterogeneities of these cancers. In this review article, we discuss new findings that are greatly improving the understanding of thyroid cancer biology and facilitating the identification of novel targets for therapeutic intervention. We review the phenotypic features of different subtypes of thyroid cancers and their underlying biology. We discuss recent discoveries in thyroid cancer heterogeneities and the critical mechanisms contributing to the heterogeneity with emphases on genetic and epigenetic factors, cancer stemness traits, and tumor microenvironments. We also discuss the potential relevance of the intratumor heterogeneity in understanding therapeutic resistance and how new findings in tumor biology can facilitate designing novel targeting therapies for thyroid cancer.

## 1. Introduction

Thyroid cancer represents approximately 95% of all endocrine tumors, accounting for roughly 2.5% of all malignancies. In 2020, the incidence of thyroid cancer in the United States was estimated to be 52,890 cases, and approximately 2180 patients (4.1%) were expected to die from thyroid cancer [1]. Thyroid cancer incidence has been steadily rising in recent years (approximately 4% increase annually), and represents the most rapidly increasing malignancy among all major cancers in the United States, tripling in the past three decades [2]. Ultrasound, which enables detection of small thyroid nodules that might not otherwise have been discovered in the past, has greatly improved the diagnosis and the subsequent number of thyroid cancer cases. Important risk factors of thyroid cancer include a history of exposure to radiation and a family history of thyroid cancer. High-risk patients were elder (female, >51 years; male, >41 years) with distant metastases, with signs of adjacent structure invasion, or with a primary tumor in diameter greater than 5 cm [3].

Most carcinomas in the thyroid are well-differentiated tumors originated from follicular cells, defined as papillary thyroid carcinoma (PTC) or follicular thyroid carcinoma (FTC). Around 79% of thyroid cancer cases are papillary carcinoma, and 13% are follicular carcinoma. Another entity of well differentiated thyroid tumors, which are no longer seen as a variant of FTC, is Hürthle cell carcinoma (HCC), representing approximately 5% of all thyroid malignancies arising from follicular cells. Medullary thyroid carcinoma (MTC) accounts for about 4% of thyroid cancers, which arise from parafollicular C cells. Anaplastic thyroid carcinoma (ATC), an undifferentiated subtype of thyroid cancer, constitutes 2% of thyroid malignancies. Poorly differentiated thyroid carcinoma (PDTC) is a rare subtype of thyroid cancer and is placed biologically between PTC or FTC and ATC, with an incidence of around 2% to 15% of all thyroid malignancies. Despite that the overall mortality of thyroid cancer is low in an average of 0.46 per 100,000 patients due to the effective and safe therapies for most forms of the disease [4], 90% of ATC patients die within 6 months of diagnosis. ATC accounts for more than 50% of annual death related to all thyroid cancers. The high mortality of ATC is due to its significant likelihood of resistance to conventional cancer therapies in addition to its rapid growth rate as well as being detected in its most advanced stage [5].

The phenotypic heterogeneity of most cancers generally results from the integration of both genetic and non-genetic influences. Recent advances in multiscale profiling and deconvolution of tumor tissue bulk gene expression data have improved the understanding of the tumor landscape and have been leading to the potential identification of novel biomarkers and molecular targets for prognosis and therapeutic intervention. In this review, we discuss phenotypic heterogeneity and the underlying genetic and non-genetic contributing factors in thyroid cancer, and the implications of intratumor heterogeneity in understanding therapeutic resistance and developing effective strategies to target thyroid cancer.

## 2. Phenotypic Heterogeneity of Thyroid Cancer

The thyroid gland is a complex endocrine organ that gives rise to cancers differing in tumorigenicity, invasiveness, morphology, and molecular profile. The broad heterogeneity of thyroid cancer represents one of the most variable tumors among all malignancies. Phenotypic heterogeneity in tumor cell populations that results from the influence of genetic and non-genetic determinants comprises a major source of therapeutic resistance [6,7]. Here we review the phenotypic heterogeneity in subtypes of thyroid cancers.

### 2.1. Papillary Thyroid Carcinoma

PTC is the most common subtype among well differentiated thyroid carcinomas (DTC). The gross appearance of the papillary cancer is considerably variable. Generally, the lesions are firm, white, and not encapsulated. Calcifications, necroses, or cystic changes may be macroscopically apparent [8]. Historically, PTCs originate from thyroid follicular cells. The tumors consist of papillary structures that comprise neoplastic epithelia overlying an actual fibrovascular stalk. PTC rarely exists as a homogenous tumor and has numerous histopathologic variants (Table 1). The aggressive subtypes include tall cell variant (TCV) [9,10,11,12,13], columnar cell variant (CCV) [14], diffuse sclerosing variant (DSV) [15,16,17,18], and solid variant (SV) [19,20,21]. These aggressive subtypes have been associated with high rates of multifocality, extrathyroidal extension, local and distant metastasis, and recurrence [22]. 

PTC often appears as two or more anatomically separate foci, which may display heterogeneity morphologically. Kuhe et al. suggested that some cases of multifocal PTC widely separate from each other are the result of real multicentricity, while others are the outcome of intrathyroid spread of an initially sole tumor mass inside vascular spaces, frequently accompanied by multiple lymph node metastases [28]. PTC exhibits a high level of heterogeneity in its stromal cellular composition, including desmoplasia, nodular fasciitis-like changes, inflammatory myofibroblastic feature, and myxoid and amyloid formation. The desmoplastic stromal reaction seems to be an indicator of invasive behavior of PTC significantly associated with lymph node metastases [29]. The appearance of psammoma bodies, which are located in the stroma as calcified foci with concentric laminations, is generally in association with poor disease-free survival [30]. The heterogeneity of immune cells in the tumor stroma plays an important role as well in the clinical outcome as both protumorigenic and antitumorigenic roles were observed in association with different subpopulations of immune cells [31].

### 2.2. Follicular Thyroid Carcinoma

FTC represents approximately 10% of thyroid malignancies. FTC tends to be more aggressive with potentials to invade locally and metastasize distally to the bone and lungs. This tumor is less sensitive to traditional therapy. The distinction between FTC and follicular adenoma is based on the presence of capsular or vascular invasion. FTC shows less phenotypic heterogeneity than PTC, involving microfollicular or solid growth of colloid containing follicles [32]. The presence of an insular component in FTC is an independent factor for distant metastasis [30]. The extensive capsular and vascular invasion is associated with higher metastatic potential and mortality rate [32]. 

### 2.3. Hürthle Cell Carcinoma

HCC is a subtype of well differentiated thyroid cancer, accounting for roughly 5% of thyroid cancer diagnoses [33]. The tumors arise in the thyroid follicular cells. Tumor cells are defined in part by an accumulation of abnormal mitochondria, prominent nucleoli, and a loss of cell polarity [34]. Although being long considered as a subtype of FTC, the cancer is now generally considered a separate entity [35]. Based on the degree of vascular invasion, HCCs are categorized as minimally invasive with <4 foci of vascular invasion or as widely invasive with ≥4 foci [36]. The widely invasive HCC exhibits aggressive behaviors, is prone to metastasize early, and accounts for most deaths attributable to the tumors. HCCs are refractory to radioactive iodine and unresponsive to cancer treatment agents commonly used in chemotherapy [37,38].

### 2.4. Medullary Thyroid Carcinoma

MTC arises from parafollicular C cells. The tumor may secrete calcitonin, carcinoembryonic antigen, prostaglandins, and serotonin [39]. Most MTCs are present with spontaneous unifocal lesions in patients 50 to 60 years old (sporadic form). The remaining cases (about 30% of all MTCs) affecting young individuals are hereditary with autosomal dominant traits. At least 35% of MTC patients have lymph node metastases at diagnosis, while approximately 20% of cases have distant metastases. Multiple endocrine neoplasia (MEN) type IIA and IIB syndrome are associated with MTC [40]. MTCs are comprised of neoplastic cells that are heterogeneous both in shape and size. The tumors contain collagen, amyloid, and dense irregular calcification, which are heterogeneous, separating the neoplastic cells. The heterogeneity of MTC may present as merger of MTC and PTC with both components being intermixed and may exhibit morphological features of both subtypes within the same lesion. Moreover, the cervical lymph node metastasis generally displays both subtype components as a mixture within the same lymph node [41].

### 2.5. Poorly Differentiated Thyroid Carcinoma

PDTC is a rare subtype of thyroid cancer with an incidence reported as 2% to 15% of all thyroid malignancies while accounts for most fatalities in follicular cell-derived non-ATC cancers [42]. The incidence variation is likely the indication of geographic influences or differences in interpretation of histopathological findings [43]. Tissue pathology, as well as clinical behavior of PDTC, is commonly considered as an intermediate within thyroid follicular cell-derived tumor progression model, standing between differentiated thyroid cancer and ATC. Most PDTCs arise in well-differentiated PTCs or FTCs, although a subset of PDTCs apparently arise de novo [44]. PDTC is generally diagnosed according to the Turin-PDTC criteria [45]. It has been well known that PDTC can have minor and even major components of a well-differentiated thyroid cancer like PTC or FTC. Unlike differentiated thyroid cancer, PDTC tends to present with locally extrathyroidal invasion in more than 50% of patients [46,47], metastasizes to regional lymph nodes in up to 50%–85% of cases [42,48] and to distant locations in up to 85% of cases [48], and has a high tendency of local recurrence after surgical treatment [49].

### 2.6. Anaplastic Thyroid Carcinoma

ATC is one of the most aggressive tumors, with disease-specific mortality approaching 100% [50,51,52]. These tumors affect patients approximately 60 to 70 years of age. Of these malignancies, 80% may occur with a coexisting carcinoma and may be transformed from a well-differentiated form of thyroid cancer [53]. ATCs are the most heterogeneous tumor of all the thyroid cancer subtypes. Microscopically, ATCs present a broad spectrum of differentiation, consisting of mixtures of pleomorphic giant cells and epithelioid cells as well as spindle cells [54]. The stroma of ATC shows variable hyaline, sclerosis, or desmoplasia. 

## 3. Genetic Heterogeneity of Thyroid Cancer

Thyroid cancer heterogeneity is not only limited to phenotypic diversity but also manifested as variation in genetic alternations. Genomic analyses revealed complex mutations with vast intertumor and intratumor heterogeneity. Intertumor heterogeneity refers to genetic variants occurring among individuals with the same tumor type. Intratumor heterogeneity refers to a subclonal diversity within the same tumor lesion, including a process that transforms a tumor into a more aggressive cancer through additional genetic alterations. Cancer initiation and progression are reliant on the acquisition of multiple driver mutations that either activate oncogenic pathways or inactivate tumor suppressors. Thyroid cancer represents a type of neoplasia in which critical genes are often mutated through two different molecular mechanisms: point mutation (BRAF, RAS, TP53, and CTNNB1) and chromosomal rearrangement (fusion of the RET gene to several unrelated genes known as RET/PTC rearrangement, RET/PTC) [55,56]. The former is a consequence of single nucleotide changes within the DNA chain, while the latter specifies a large-scale genetic aberration with breakage and fusion of parts of the same or different chromosomes. Increasing evidence suggests that these two individual mutational mechanisms are linked to specific etiologic factors associated with thyroid carcinogenesis. Such genetic alterations further affect downstream signaling pathways, including MAPK (mitogen-activated protein kinase, MAPK), PI3K/AKT (phosphatidylinositol-3' kinase, PI3K/AKT), and mTOR (mammalian target of rapamycin, mTOR) pathways [57]. Because tumor cells accumulate large numbers of genetic mutation, closer attention should be given to potential interactions among these genetic mutations and signaling pathways. The main pathways and mutations that are involved in thyroid oncogenesis are described as following. Since this section focuses on the most dominant pathways and mutations in thyroid cancer in general, we refer researchers who are interested in detailed PTC-specific genetic events about intratumoral heterogeneity to a review article by Fugazzola et al. [58].

### 3.1. MAPK Pathway

The MAPK signaling pathway is one of the most extensively investigated pathways in oncology and plays a crucial role in thyroid carcinogenesis. Upon pathologic activation of different cell-membrane receptor tyrosine kinases, a cascade of downstream events subsequently occurs, which ultimately causes aberrant cell proliferation, differentiation, and survival. According to the data from the cancer genome atlas, PTC has been classified as a MAPK driven tumor with two significant signaling drivers—mutated *BRAF* and *RAS* [59]. These two distinctive mutations occur in approximately 70% of PTC patients and are linked to particular biological, histopathological, and clinical characteristics of the tumor [60]. *RAS* genes encode highly related G-proteins. Such proteins are located in the inner surface of the cell membrane and able to transmit signals arising from receptor tyrosine kinases of the cell membrane and from G-protein coupled receptors along signaling pathways of MAPK, PI3K/AKT, and others. *RAS* mutations were detected in a variety of thyroid cancers, including 10–20% of PTC, 40–50% of FTC, 10% of HCC, and 20–40% of PDTC and ATC [61,62,63]. *BRAF,* a serine-threonine kinase, can translocate to the cell membrane once being bound and activated by *RAS.* The translocation leads to phosphorylation and activation of MAPK kinase and the kinase signaling pathway downstream targets. The most common mechanism of *BRAF* activation in thyroid cancer involves a point mutation that comprises a substitution of thymine to adenine at nucleotide position 1799, which results in replacement of a valine-to-glutamate at residue 600 (Val600Glu) [64]. The *BRAF*^Val600Glu^ is the most frequent genetic variation in PTCs (40–45%). It is typically detected in the classic papillary and tall-cell variants but is rare in the variant of follicular histology [60]. A recent study reported that *BRAF*^Val600Glu^ might facilitate decision-making on active surveillance of PTC [65]. In addition, two multi-institutional studies found that *BRAF*^Val600Glu^ confers male sex disease-specific mortality risk in PTC patients and that PTC patient age-associated mortality risk can be differentiated by *BRAF*^Val600Glu^ status [66,67]. The mutation is also found in 20–40% of PDTC and 30–40% of ATCs [68,69]. Many of the PDTC and ATC carcinomas also reveal areas of well-differentiated phenotypic cancer, and *BRAF*^Val600Glu^ is found in both tumor components, which suggests that this mutation predisposes the tumor to dedifferentiation and represents an early event in thyroid cancer development.

RET/PTC is a chromosomal rearrangement with a portion of the *RET* gene fused to one of several possible partner genes. Those genes encode the intact tyrosine kinase domain fused to an active promoter of a different gene that can drive the expression and dimerization of the RET/PTC protein in ligand independent manners, which consequently ends with chronic stimulation of MAPK signaling and thyroid tumorigenesis [70]. The prevalence of RET/PTC rearrangements in PTCs varies dramatically in reported series of patients, which is likely due to the heterogeneous distribution of this rearrangement within the carcinoma tissues and the diverse sensitivities associated with the detection methods. Clonal RET/PTC rearrangement occurs in 10–20% of PTCs and is specific for this subtype tumor [71].

### 3.2. PI3K/AKT

PI3K/AKT pathway is a very critical molecular signaling pathway that can be activated by some aberrant receptor tyrosine kinases (RTKs). Genetic mutations or ligand mediated downstream effector activations will lead to high levels of cell proliferation in a wide range of cancers, including the cancers in the thyroid. PI3K can be activated in thyrocytes by insulin / insulin-like growth factors (IGF), epidermal growth factor, or hepatocyte growth factor. The important pathways mediating PI3K signaling are PI3K/AKT/mTOR and PI3K/AKT/PTEN (phosphatase and tensin homolog, PTEN) signaling pathways.

The mTOR (also named for FRAP1) is considered an essential regulator of cell growth. mTOR is a serine/threonine kinase; as a molecular sensor, it controls protein synthesis in response to diverse signals, including different mitogens, growth factors, and nutrients. The dysfunction of mTOR has been witnessed in various human cancers [72]. mTOR regulates biogenesis by activating p70 S6 kinase (p70S6K, also called ribosomal protein S6 kinase beta-1) and inhibiting 4E binding protein 1 (4EBP1), which subsequently ends with an increase in the synthesis of protein and biogenesis of ribosomes [72]. Evidence showed that the co-expression of mTOR complex 1 (mTORC1) and mTOR complex 2 (mTORC2) commonly occurs in PTCs, and that simultaneous targeting of mTORC1 and mTORC2 activity could be a therapeutic strategy effectively treating PTCs [73]. Human PTCs exhibited elevated levels of p70S6K, a major mTOR target, and associated with higher mTOR activity [74]. Constitutive activation of the mTOR pathway was identified in thyroid carcinomas as compared to the tissues of normal thyroid [75,76,77]. Thus, identification of the role in thyroid tumorigenesis played by the mTOR pathway is worthwhile as mTOR inhibitors have been developed into potential pharmaceutical agents. In fact, the RET proto-oncogene mutations have been recognized as responsible for the pathogenesis of several forms of MTC [76,78]. The PI3K/AKT/mTOR pathway deregulation is found to play important roles in the tumorigenesis induced by RET proto-oncogene mutations. Based on these observations, it is interesting to investigate whether targeting the PI3K/AKT/mTOR pathway at simple or multiple sites with specific inhibitors could be an attractive approach for the potential treatment of patients with advanced MTCs as indicated previously [79].

The tumor suppressor gene *PTEN* is located on chromosome 10. Its mutations or loss of function are frequently found in both heritable and spontaneous cancers [80]. PTEN represents a negative regulator in the PI3K signaling, and its dysfunction can dramatically affect the PI3K pathway. *PTEN* mutation in germline cells results in Cowden’s syndrome (CS), an autosomal dominant syndrome that displays an association with a higher risk of a variety of cancers, including breast, endometrial, and thyroid cancers [81]. Around two-thirds of the CS patients are diagnosed with thyroid malignancies, in which FTCs are identified as the most common thyroid cancer [82]. *PTEN* mutations are uncommon in sporadic thyroid cancers, especially among the differentiated types [17,79,80,81]. The FTC has a higher probability of mutation and methylation in *PTEN* than the PTC [79,80,81]. Among the thyroid cancers, ATCs have the highest rate of *PTEN* deletion and mutations, and these tumors are expected to carry many genetic changes [82].

### 3.3. Insulin/IGFs

IGFs are small ligands (~7.5 kDa) that play an important role in various biological processes, including cell proliferation, apoptosis protection, and somatic growth and development. The functions of insulin, IGF-1, and IGF-2 are mediated through the RTKs insulin receptor (IR) and type 1 IGF receptor (IGF-1R) located on the cell surface [3], resulting in multiple downstream signaling pathways activation, including the MAPK and PI3K/AKT pathways [83]. These pathways can also be activated by other RTKs, including the receptors for epidermal growth factor, fibroblast growth factor, and hepatocyte growth factor. It is known that complex cross-talk exists between these pathways and the IGF axis [84]. Evidence suggests that thyroid cancer cells and precursors of the cancer cells are responsive to the activity of insulin and IGFs, and frequently overexpress IR and IGF-1R during the early stage in thyroid carcinogenesis [85]. In fact, most thyroid cancer cells overexpressed IR, which can amplify the biological effects of IGF-1 through forming hybrid IR/IGF-1R receptors that will bind IGF-1 with high affinity. Moreover, IR overexpression may directly enhance the response of thyroid cancer cells to IGF-2 by the activation of IR-A, a newly recognized autocrine loop, functions as a high affinity IGF-2R [85]. The IGF-2/IR-A loop activation has a recognized role in tumor progression, dedifferentiation, and therapeutic resistance. The IR-A overexpression is a feature of anaplastic or cancer stem-like thyroid cancer cells [86]. In contrast, IGF-1R expression decreases with cancer dedifferentiation, suggesting that the IGF-2/IR-A loop plays a more important role than does the IGF-1/IGF-1R loop in thyroid cells dedifferentiation, the acquisition of a stem-like phenotype, tumor progression, and metastasis [87].

### 3.4. PAX8/PPARγ Rearrangement

PAX8 drives the expression of numerous thyroid specific genes that encode thyroglobulin, the sodium iodide symporter, and the thyroid peroxidase. PAX8/PPARγ (peroxisome proliferator-activated receptor gamma, PPARγ) rearrangement results in the fusion between a portion of the PAX8 gene, which encodes a paired domain transcription factor, and the PPARγ gene. The fusion leads to the chimeric PAX8/PPARγ protein in strong overexpression [88]. PAX8/PPARγ rearrangement is a prototypic change found in follicular thyroid carcinoma (30–35%). This rearrangement is also identified in follicular adenomas (2–13%) and in a minor proportion of the follicular variant (1–5%) of papillary carcinomas [89], as well as in HCC [90] and noninvasive follicular neoplasm with papillary-like nuclear features (NIFTP) [91]. The overlap of PAX8/PPARγ rearrangements and *RAS* point mutations rarely happens in the same tumor, suggesting that they represent distinct pathogenetic pathways in the event of FTC tumorigenesis [56].

### 3.5. Widespread Chromosome Loss and mtDNA Mutation

Widespread chromosomal losses represent a hallmark of HCC. Corver et al. initially reported a widespread loss of chromosomes in a series of HCCs [92], and subsequently, Kasain et al. uncovered evidence for extensive chromosome loss in another study using whole-genome sequencing [93]. Recent studies by Ganly et al. [94] and Gopal et al. [95] identified the widespread chromosomal loss as a common feature for HCC in larger tumor cohorts. Along with chromosomal loss, it was found that a complex or polysomic genotype due to whole-genome duplication was observed frequently in widely invasive HCCs [94]. Despite that two copies of the haploid chromosomes were restored, the duplicated copies arose from a single parent chromosome, which is called in a term “uniparental disomy” that consequently resulted in widespread loss of heterozygosity (LOH) in HCC. Widespread LOH was found to be associated with a poor prognosis [94,95], suggesting that widespread chromosomal losses contributed to more aggressive behaviors of the disease.

Interestingly, the integration of transcriptomic and mutational data identified intertumoral heterogeneity and led to the classification of HCC into three subtypes [94]. The first subtype consisted of widely invasive HCC with TERT (telomerase reverse transcriptase, TERT) alterations, widespread LOH, and chromosome 7 amplification. Disease recurrences and deaths occurred mostly in this group. The second subtype included tumors with no TERT alterations but harboring widespread LOH and chromosome 7 amplifications. This group presented low frequencies of disease recurrence and death. The last subtype included minimally invasive HCC without presentation of TERT alterations, LOH, or chromosome 7 amplification. No disease recurrences or deaths were observed in this group. If these findings being validated with additional patient cohorts, the integration of transcriptomic and genomic mutational data might assist in the clinical identification of patients with potentials of high risk in disease recurrence and thus guide treatment decision planning for these patients.

The remarkable abundance of dysfunctional mitochondria in the cancer cells (>75% of cell volume) has been considered as the most noteworthy feature of HCC [96]. Recent studies by Gopal et al. [95] and Ganly et al. [94] performed mtDNA (mitochondrial DNA, mtDNA) analyses with large panels of HCCs. There were three important discoveries involving mtDNA mutations reported in their studies. First, consisting of the findings reported previously by Gasparre et al. [97], mutations of mtDNA were particularly enriched in genes encoding the complex I components of the mitochondrial respiratory chain. Second, mtDNA mutations, particularly disruptive mutations in complex I, appeared more frequently in HCCs compared with other cancers [98]. Third, if appeared, the disruptive mtDNA mutations presented in the majority of mitochondria within HCCs. In addition, disruptive mutations in the complex I were sustained in metastases when the primary HCCs had the mtDNA mutations. These observations suggest that disruptive complex I mutations might act as HCC-driving events.

### 3.6. Telomerase Reverse Transcriptase Promoter Mutations

TERT promoter gain-of-function mutation is particularly important in thyroid cancer pathogenesis [99,100,101]. The gain-of-function mutation creates a de novo ETS (E26 transformation-specific, ETS)-binding motif through which TERT transcription is de-repressed, and telomerase is activated [101]. Telomerase activation has been shown as the most common strategy for thyroid tumor cell infinite proliferation [99]. TERT promoter mutations occur predominantly at two sites with a cytidine-to-thymidine (C>T) dipyrimidine transition, C228T and C250T, in the proximal region of the promoter (−124 and −146 bp from ATG, respectively) [102,103]. These mutations were initially reported in thyroid cancer by Liu and coauthors [104]. The study demonstrated that the promoter mutation frequency was 12%, 14%, 38%, and 46% in PTCs, FTCs, PDTCs, and ATCs, respectively [104]. Consistent with the initial findings, another report showed that the mutations occurred in 17–22% of FTCs, 25% of PTCs, and 50% of ATCs [105]. Landa et al. also observed similar mutation frequencies in the TERT promoter [106]. Up to now, around 200 research articles have been published, which involved more than 15,000 patients who were subjected to analyses for thyroid cancer TERT promoter mutation statuses and clinical significance. Based on these analyses, the following findings were concluded: (I) tumor aggressiveness and TERT promoter mutation rates were intimately correlated. The most aggressive thyroid cancers, including PDTCs and ATCs, exhibited the highest frequencies of TERT promoter mutations; (II) C228T was found as the predominant mutation, and the presence of C228T and C250T was generally mutually exclusive. In rare cases, C228T and C250T could occur in different subclones or different cell subpopulations within the same tumor, demonstrating intratumor heterogeneity; (III) young thyroid cancer patients (<45 years old) rarely had the mutation, especially in pediatric cases; (IV) there were ethnic and geographic differences in TERT promoter mutations. 

Interestingly, it was recently reported that the transcription factor FOS was phosphorylated and activated by the BRAF^Val600Glu^-mediated hyperactive MAPK signaling [107]. The activation of FOS facilitated the transcription of GABPB1, a member of the ETS transcription factor family, thereby enhancing TERT expression in thyroid cancer cells bearing mutant TERT promoter [107]. These findings provided evidence of a functional link between BRAF^Val600Glu^ and mutant TERT promoter and a possible reason for why the two genetic events were frequently co-existing in PTCs.

PTCs with TERT promoter mutations expressed TERT [107,108,109] and might demonstrate high levels of TERT expression and telomerase activity when PTCs were transformed into ATCs [110]. TERT can serve as a transcription co-factor, which interacts with a panel of transcription factors facilitating the target transcription essential for oncogenesis [111,112,113]. In addition to conferring cancer cells an immortal phenotype, TERT empowers the cells with stemness, invasiveness, drug resistance, and anti-apoptotic ability [99,111,112,113,114,115,116]. Thus, TERT promoter mutation-induced TERT can exert oncogenic functions via both telomere-dependent and telomere-independent mechanisms, thereby promoting the development and progression of thyroid cancer.

Investigations of clinical thyroid cancer patients have revealed that TERT promoter mutation is highly correlated with advanced or progressive disease, including advanced tumor stages, extrathyroidal extension, vascular invasion, lymph node metastases, distant metastases, and recurrence [100,101,104,105,110,114,117]. Evaluating TERT promoter mutations and patient outcomes has demonstrated that the mutation can be an independent factor in predicting thyroid cancer shorter disease-free survival [114,118,119,120,121,122,123,124,125,126,127]. Although BRAF^Val600Glu^ as a tumor driver event alone may not significantly affect patient outcome, its co-existence with the TERT promoter mutation results in the formation of aggressive PTC and even promotes the evolution of PTC to poor-differentiated tumors or ATCs [118,119].

### 3.7. Mutations in Tumor Dedifferentiation

Anaplastic and poorly differentiated thyroid cancers are frequently having additional genetic alterations that are not found in well-differentiated ones. Such genetic alternations represent late events and may be essential for the initiation of tumor dedifferentiation. These late events comprise the *TP53* and *CTNNB1* mutations, as well as mutations in genes that encode downstream effectors in the signaling pathway of the PI3K/AKT. The *TP53* gene (which encodes the cell cycle regulator p53) has point mutations that are detected in a major proportion of anaplastic carcinoma cases (50%–80%) [128]. Such mutations are extremely rare in well-differentiated thyroid cancers. The mutations result in this important tumor suppressor gene being a loss of function. *CTNNB1* represents another frequently mutated gene in anaplastic carcinoma, which encodes a molecule involved in cell adhesion and Wnt signaling called β-catenin. The *CTNNB1* gene has point mutations in exon 3 found in more than half of anaplastic carcinomas (up to 60%). These mutations are also identified in other poorly differentiated thyroid carcinomas, although in lower prevalence [129].

**Figure 1:**
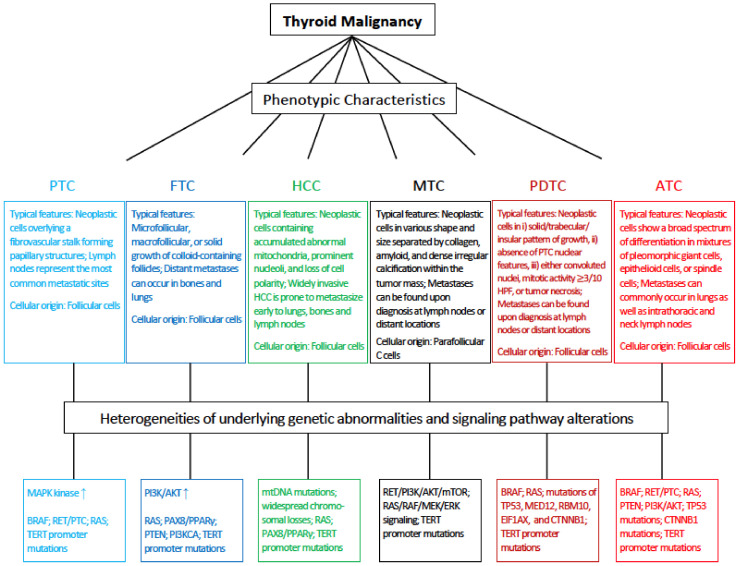
Overview of the phenotypic characteristics and underlying mechanisms of thyroid carcinomas. There are five histologic types of thyroid cancers with variably morphological features. Heterogeneous genetic abnormalities and signaling pathway alterations contribute to the phenotypic variations. PTC: papillary thyroid carcinoma; FTC: follicular thyroid carcinoma; HCC: Hürthle cell carcinoma; MTC: medullary thyroid carcinoma; PDTC: poorly differentiated thyroid carcinoma; ATC: anaplastic thyroid carcinoma; HPF: high power microscopic fields; MED12: mediator complex subunit 12; RNA-binding motif 10; EIF1AX: Eukaryotic Translation Initiation Factor 1A X-Linked.

With an increased understanding of genetic alterations in thyroid cancers (Figure 1), strategies targeting the alterations are becoming potential attractive therapies for the disease. While some clinical benefits have been achieved with multiple kinase inhibitor drugs (MKIs) targeting the MAPK pathway, the development of tumor escape mechanisms to these drugs signifies additional limitations besides their systemic toxicity [130]. Among the pathways involved in thyroid cancers, there are overlaps among the MAPK and PI3K/AKT cascades, with upregulation of either individual pathway leading to a similar end-result as tumor cell survival and proliferation. The essential components of MAPK pathway, RAS and RET/PTC oncogenes, have cross-talk effects with the cascade of PI3K/AKT. In light of these facts, strategies exploiting multiple pathways targeting agents are likely to produce improved therapeutic effects in the treatment of thyroid cancers.

## 4. Non-Genetic Heterogeneity of Thyroid Cancer

Although genetic heterogeneity is considered a general feature of human cancers, current evidence does not support that tumor phenotypic diversity is merely a product of genetic heterogeneity. For example, genetically homogenous cell populations show remarkable diversity in terms of their response to therapy [131], suggesting that other non-genetic sources of heterogeneity also play important roles. 

### 4.1. Cancer Stem Cell Heterogeneity

Cancer stem cell (CSC) research has promoted the dominant framework for identifying non-genetic sources of phenotypic heterogeneity in tumor cells [132]. The CSC hypothesis was initially validated in human leukemia based on the findings that a small fraction of tumor cells were able to generate leukemia in severe combined immunodeficient mice, while the vast majority of tumor cells were unable to engraft and cause tumor [133]. Subsequently, CSCs were isolated from solid tumors [134,135,136,137,138], which are typically heterogeneous at the functional as well as molecular level. The CSC model provides an elegant explanation for cancer cell phenotypic and functional heterogeneity in most tumors. The model suggests that cancers are hierarchically organized into subpopulations of tumorigenic CSCs and their non-tumorigenic progenies. In other words, only CSCs, as a small fraction of the whole population of tumor cells, are believed to drive tumor growth and disease progression, likely by uncontrolled self-renewal, therapy resistance, and metastasis initiation [139]. 

A large body of evidence from CSC research has demonstrated that only a subset of cancer cells possesses the ability to self-renewal and maintains tumor growth. CSCs generate cellular heterogeneity by installing a hierarchical differentiation leading to a range of distinct cell types present within the individual tumor [140]. Studying thyroid cancer stemness, different markers/methods have been used in isolating CSCs from clinical tumor specimens as well as cancer cell lines (Table 2) [87,141,142,143,144,145]. They include flow cytometry sorting based on cell surface expression of CD44 [142] and CD133 [145], identification of side-population phenotypes through Hoechst 33342 exclusion [141], isolation of the cytoprotective enzymes aldehyde dehydrogenase (ALDH) positive cell fraction [143], and sphere-forming cell isolation with serum-free culture [87]. CSCs isolated with these strategies are generally heterogeneous populations. Specific signaling pathways have been indicated as important players functioning in the heterogeneous CSCs. These pathways, including Notch, Wnt/β-catenin, and hedgehog (HH), have been proved to participate in the regulation of self-renewal and survival in normal stem cells. They have been analyzed in CSCs of various cancer types, including those of thyroid cancers. 

Notch signaling pathway has been implicated in self-renewal and differentiation of CSCs in a variety of cancers. The expression of Notch receptors has been observed during the thyrocyte development in experimental systems, and the expression levels of these receptors are aligned with the markers representing thyroid cell differentiation [146]. There are studies demonstrating that Notch-1 overexpression in thyroid cancer cells restores differentiation, stimulates the expression of differentiation markers, and reduces cell growth [147]. The expression levels of Notch-1 were downregulated in thyroid cancer tissues compared with thyroid tissues of benign. Decreased Notch-1 was associated with a higher recurrence rate and extrathyroidal invasion [148]. Restoration of Notch-1 function reduced cell growth and migration in a doxycycline-inducible metastatic DTC cell line, as well as significantly reduced the primary tumor growth and inhibited the lung metastasis development in a thyroid cancer model [148]. Furthermore, the Notch-1 receptor may represent a predictor of lymph node metastasis and may serve as one of the poor prognostic markers in PTC patients [149]. Interestingly, Kim et al. demonstrated that the activated Notch-1 expression was higher in cases of ATC than in cases of PTC. Inhibiting Notch-1 significantly reduced the proliferation and migration of ATC cells, but not that of PTC cells [150]. In addition, inhibiting Notch-1 in ATC cells drastically reduced the expression of key markers representing epithelial to mesenchymal transition and cancer cell stemness. Conversely, changes in the expression of these markers were not observed in PTC cells when the Notch-1 inhibition was applied [150]. This study demonstrated the different roles of Notch-1 expression in cancer cell aggressiveness between ATC and PTC, suggesting the heterogeneity of CSCs in thyroid cancer.

Wnt pathway activation causes the accumulation of β-catenin in the cell cytoplasm, eventually translocation into the nucleus and acting as a transcriptional co-activator of the T cell factor/lymphoid enhancer factor (TCF/LEF) family of transcription factors. This pathway controls many processes of cellular and developmental biology, including cell proliferation, cell fate determination, and tissue homeostasis. Given its critical roles in the maintenance of stem cell survival [151], the Wnt signaling pathway has been implicated in normal thyroid development, homeostasis, and carcinogenesis [152]. Using reverse phase protein microarray technology and immunoblot validation, Todaro et al. revealed a considerable upregulation of β-catenin in sphere cells from ATC as compared with those from normal thyroid, PTC, and FTC [143]. In addition, ATC sphere cells showed a higher percentage of β-catenin nuclear accumulation [143]. These observations suggest a possible role of Wnt pathway in the aggressiveness of thyroid CSCs.

The HH pathway is evolutionarily conserved and plays an essential role in the embryonic normal tissues and organs development, as well as in tumorigenesis. The HH pathway is highly activated in more than 65% of thyroid neoplasm tissues and in most thyroid cancer cell lines [153]. HH and Gli1 (a member of the Gli family transcription factors that mediate the HH signaling activity) are detected in two-thirds of PTCs and in most of ATCs [153]. Inhibition of the HH/Gli pathway by HH or Gli1 knockdown or by using Gli1 inhibitors significantly diminishes cell proliferation [153]. Bian et al. found that the expression of sonic HH (SHH)/Gli-1 was particularly associated with PTC tumor size, clinical staging, and local lymph node metastasis, indicating that aberrant activation of the HH pathway is critical to PTC disease progression [154]. Moreover, the components of the HH pathway are highly expressed in ATCs, MTCs, and in their respective cell lines [155,156]. The HH pathway is also involved in maintaining CSC pools in various tumors, including glioblastoma [157], multiple myeloma [158], myeloid leukemias [159], colorectal cancers, and gastric cancers. Suppression of the HH by knocking down HH or Gli1 expression in KAT-18 (an ATC cell line) leads to decreased number and size of thyrospheres, whereas overexpressing Gli1 leads to increased number and size of thyrospheres [160]. Williamson et al. showed that suppression of the HH pathway by microRNAs (miRNAs) that target either HH or Gli1 in KAT-18 cells decreased motility and invasiveness of the cells [161]. By contrast, Gli1 overexpression in the KAT-18 cells reversed the effects [161]. Together, these observations suggest that the activation of the HH pathway stimulates the thyroid CSC motility and invasiveness. 

### 4.2. Epigenetic Heterogeneity

Epigenetics is defined as gene expression changes heritable without alterations in DNA sequence. Several epigenetic mechanisms involving DNA methylation or miRNA interference play diverse roles in cancer by promoting, sustaining, enhancing, or inhibiting malignant phenotypes at various stages of the diseases. Aberrant DNA methylation is associated with alterations in gene expression and plays a critical role in tumorigenesis [162]. DNA hypomethylation leads to genomic instability and proto-oncogenes activation through diverse mechanisms, which contribute to tumor initiation and progression, while DNA hypermethylation is associated with gene silencing, specifically tumor suppressor genes, and it is believed to be the hallmark of malignancies [163]. DNA methylation of tumor suppressor genes is common in thyroid carcinomas. Certain tumor suppressor genes specifically in the thyroid are found as *PTEN, TIMP3, SLC5A8, DAPK, RAPβ2,* and *RAP1GAP*.

*PTEN* is a tumor suppressor gene and has been found to be mutated in many cancers. PTEN negatively regulates the AKT signaling pathway, and it involves in the cell cycle control, consequently preventing excessive cell growth and rapid division [164]. Aberrant DNA methylation of *PTEN* was detected in 50% of PTC and in all cases of FTC [165]. TIMP3 is a tissue inhibitor of metalloproteinase and is able to inhibit tumor angiogenesis, tumor growth, invasion, and metastasis of several malignancies [166]. This gene hypermethylation has been observed in thyroid cancers (53% of PTC), and the association of the gene with extrathyroidal invasion and lymph node metastasis has been reported [167]. The *RAP1GAP* gene encodes a type of GTPase-activating protein. The significance of the gene has been implicated in regulating the thyroid cell mitogenic and oncogenic pathways, which is mainly mediated by downregulating the activity of the RAS-related protein [168]. Aberrant DNA methylation of this gene was found in 72% of PTC and in 38% of FTC [169]. In addition, the identification of 280 and 393 hypomethylated genes while 86 and 131 hypermethylated genes in ATC and MTC, respectively, was reported. Among the identified genes, hypomethylation was the regulation frequently observed in four oncogenes (*INSL4, DPPA2, TCL1B*, and *NOTCH4*) [170]. 

Another epigenetic modification involves miRNA regulation. miRNAs are referred to as noncoding RNA molecules that are small and function as gene expression negative regulators by binding to the candidate mRNAs at the 3´-untranslated region, causing a translation blockade or degradation of target mRNAs that involve various pathophysiological events [171]. miRNA regulation of classical oncogenes and tumor suppressor genes was recognized as a hallmark in the field of cancer research, which focuses on the translation of this class of small RNAs into potential applications for cancer diagnosis, prognostic evaluation, and therapy. Recent findings in miRNA deregulation studies have revealed aberrant miRNA (miR-222, miR-221, and miR-146b) expression increase in PTC tumor tissues in comparison with normal thyroid tissues [172]. Similarly, miRNAs have been found abnormally expressed in ATC tissues and cells compared with that in non-neoplastic thyroid tissues and cells [173]. A believed target of these miRNAs was assumed to be c-KIT that functions as a tyrosine kinase receptor and plays a critical role in cell differentiation and growth. c-KIT expression is frequently found in benign thyroid adenomas and goiter, while its expression is reduced in about 60% of FTC and is entirely absent in ATC and PTC [174]. Some studies showed that miRNAs play a crucial role in MTC biology and represent a significant class of biomarkers and targets for MTC prognosis and therapeutic intervention [175]. miR-9 has been identified as a biomarker specific for sporadic MTCs. The miR-9 expression is recognized as lower in sporadic MTCs compared to heritable ones [176]. Overexpression of miR-183 and miR-375 in MTC have been suggested as important biomarkers for the prediction of lateral lymph node metastases [176]. Overall, emerging evidence in cancer epigenetic studies has revealed that genome packaging is as important as the genome by itself in the processes of regulating essential cellular events. Understanding the epigenetic landscape of thyroid cancers and its contribution to thyroid cancer heterogeneity requires further investigations.

### 4.3. Effects of Heterogeneous Tumor Microenvironment

Tumor progression involves dramatic tumor microenvironment changes, which include alterations in the properties of stromal cells and remodeling of the extracellular matrix (ECM) [177,178]. Tumor cells are constantly exposed to aberrant microenvironments, such as contracting directly with abundant fibroblasts, inflammatory cells, and remodeled ECM, which are heterogeneous among individual tumors. The expression of cancer-associated fibroblasts (CAF)-related proteins in cancer cells and stromal cells of PTCs were different according to histologic subtype, BRAF mutation status, and subtype of stroma, and such expression was associated with overall patient survival [179]. In a multivariate analysis, CAFs were identified as the independent risk factor for predicting lymph node metastasis in PTCs [180]. There is evidence that different immune cells, such as macrophages, mast cells, neutrophils, and lymphocytes, are playing pro-tumorigenic roles in thyroid cancer [31]. Abnormal microenvironments can have pronounced impacts on epigenetic landscapes of cells, translating cells into different phenotypes through epithelial-mesenchymal transition (EMT) [181]. EMT is a biological process in which epithelial cells can be transformed to a mesenchymal phenotype via multiple biochemical changes. The transformed cells are able to acquire a multipotent stem-like cell phenotype in pathophysiological conditions [182]. The reverse process, termed mesenchymal-epithelial transitions (MET), is the conversion of mesenchymal cells to epithelial derivatives. Both processes are considered to be particularly important in the development of the tumors and local or distant metastasis [182]. The molecular basis of EMT/MET regulation is complex and still poorly understood, especially for thyroid cancer. One of the characteristics of the EMT process is the loss of epithelial markers, such as E-cadherin and cytokeratin, and acquisition of vimentin, fibronectin, or neural cadherin (N-cadherin), which are commonly identified in cells of mesenchymal phenotype. The alterations in the pattern of E-cadherin and N-cadherin expression are found to be associated with resistance to anoikis, facilitation of angiogenesis, and thus promoting metastasis [183]. The specific cellular proteins in the EMT of thyroid cancer are reviewed as follows.

E-cadherin expression is found in normal thyroid tissues and benign thyroid lesions, while such expression is reduced with the transformation from differentiated thyroid cancers to undifferentiated ones [184,185]. The E-cadherin encoding gene (*CDH1*) is commonly downregulated by diverse mechanisms involving inherited and somatic mutations, abnormal protein processing, hypermethylation in *CDH1* promoter region, as well as repressing effects of Zeb, Snail, E12/E47, or Twist [186]. The expression profile of poorly differentiated thyroid carcinoma exhibits a significant reduction in E-cadherin in comparison with that of paired PTC [184]. Overexpressing BRAF^V600E^ in PTC cells induces EMT by enhancing the expression of Snail and suppressing the levels of E-cadherin [186]. The majority of ATC cases exhibited loss of E-cadherin expression, generally accompanied by overexpression of EMT-inducing transcription factors, including snail and slug [187].

Periostin, also termed osteoblast-specific factor 2, is a secreted protein that functions as a scaffold for assembling ECM proteins and has roles in the processes of cell adhesion and ECM organization [188]. Periostin can interact with integrins and thereby enhances cell adhesion and motility by modulating the AKT- and FAK-mediated signaling pathways [188]. Periostin expression is a maker and inducer of the EMT, which occurs during cancer progression. Periostin is usually upregulated in PTCs irrespective of a BRAF mutation status, but not in FTCs. The higher periostin mRNA levels are correlated with decreases in the expression of thyroglobulin and thyrotropin receptors that are thyroid differentiation markers. Also, the overexpression of periostin has been found to be associated with pathological characteristics of PTC aggressiveness, such as the extrathyroidal invasion, distant metastasis, and tumor in higher grade and stage [189,190]. These data suggest that the periostin expression is linked to thyroid tumor cell EMT activity and aggressive behaviors.

Fibronectin-1 (FN1) represents an essential ECM component. Prominent upregulation of FN1 has been found in intermediate-risk PTCs relative to the low-risk PTCs, and this finding is corroborated in metastatic lymph nodes [191]. Small interfering RNA (siRNA) mediated FN1 silencing in two different thyroid cancer cells led to a significant reduction of proliferation, adhesion, migration, and invasion in response to *BRAF^Val600Glu^* [186]. Moreover, the expression of FN1 is elevated in the PTC patients with lymph node metastasis (LNM) compared with the patients without LNM, and overexpression of FN1 is associated with PTC in larger tumor size and an advanced stage [192]. Furthermore, overexpressed FN1 is positively correlated with the diagnosis of LNM in PTC [192]. Receiver-operating characteristic analysis has demonstrated that the diagnostic value of FN1 is higher as compared to ultrasonography evaluation [192]. FN1 plays a crucial role in metastasis development in PTC by modulating the activity of tumor cell proliferation, migration, and invasion, and it is a valuable biomarker for the diagnostic prediction of LNM [192].

Vimentin is an intermediate filament protein that is expressed in mesenchymal cells. During the process of EMT, the intermediate filament and actin of the cytoskeleton can be reorganized, and cells acquire enhanced cell-matrix contacts, which promote the cell detachment from neighboring cells and trigger migration and invasion. Vimentin overexpression is associated with EMT induction in different thyroid cancer cell lines and tissues, which is correlated with several markers of mesenchymal phenotype, such as N-cadherin, Slug, Zeb1, and Snail [193,194].

S100 proteins are a group of multigene calcium-binding proteins, another family of ECM proteins related to metastasis. The upregulation of S100 proteins in the ECM can interfere with cell-cell adhesion, facilitate ECM degradation, and tumor metastasis [195]. The overexpression of S100A13, a small S100 calcium-binding protein A13, is closely associated with high intratumoral angiogenesis and poor prognosis in lung cancer and melanoma [196,197]. Enhanced S100A13 expression has been observed in PTCs as compared with normal tissues [198]. Knockdown of S100A13 has been demonstrated to diminish the expression of high-mobility group A (HMGA)-1 as well as Snail, and increase the expression of E-cadherin in thyroid cells, indicating that S100A13 may potentially play an important role in the EMT of thyroid cancer cells and the disease progression [198].

Together, multiple lines of evidence support the involvement of heterogeneous tumor microenvironments in thyroid tumorigenesis. The potential significance of tumor microenvironment heterogeneity in the enhancement of cancer stemness, cancer metastasis, and drug resistance is required to be fully unraveled.

## 5. Heterogeneity in Therapeutic Responses of Thyroid Cancer

Despite substantial advances achieved in basic and clinical cancer research, most cancers at an advanced stage remain incurable. Current therapeutic strategies for most cancer include surgical resection, radiation, chemotherapy, and second-line treatments such as hormonal or targeted therapies in an attempt to eliminate cancers by selectively killing tumor cells. If all cells within a tumor were equally sensitive to a given therapy, any therapeutic strategy that kills tumor cells quicker than the cells divide would finally lead to a cure. Unfortunately, the heterogeneity of tumor cells has prevented this from happening in most cases. Many different mechanisms at various levels limit the efficiency of current treatments for cancers. At the tumor level, cancer resistance can be primary, patients do not respond or respond very poorly to therapy because of preexisting resistant factors; or secondary, which develops after a certain period of exposure to the drug in tumors that were initially sensitive [199]. Several mechanisms that act independently or in combination are the reduction of drug influx, drug efflux pumps, alterations in drug metabolism or drug targets, activation of DNA repair mechanisms, interruptions in apoptotic signaling pathways, and enrichment of CSCs [200,201,202]. Among them, ATP-binding cassette (ABC) transporters acting as efflux pumps are the most common underlying mechanism for the evolution of resistance to different drugs [201]. Human genome codes for 49 distinct ABC transporters. Many are involved in multi-drug extrusion of toxic substances against the direction of their electrochemical gradients, which can lead to resistance of cancer cells against drugs used in chemotherapy [201].

A meta-analysis study showed that three ABC transporters, ABCB1 (multidrug resistance 1, MDR1 or P-glycoprotein), ABCC1 (multidrug resistance-associated protein, MRP1), and ABCG2 (breast cancer resistance protein, BCRP), were the most investigated transporters in ATCs [199]. ABCC1 and ABCG2 seem to be the most highly expressed transporters, and ABCB1 ranked second. They efflux anticancer drugs and, as a result, decrease the effective level of these drugs in tumor cells [199]. Doxorubicin, a common chemotherapeutic drug used in ATC treatment, is a substrate for all the main expressed ABC transporters in ATC [203]. The other ATC chemotherapy drugs (e.g., docetaxel, paclitaxel, and cisplatin) are also substrates for either ABCB1 or ABCG2 [203]. Additionally, the co-expression of different ABC transporters is a common finding in ATC, boosting the resistance to a single agent or resulting in multidrug resistance to different compounds. The high failure rate of chemotherapy in ATC could be partly explained by these findings. Several studies have demonstrated that ABCG2, ABCB1, and ABCC1 are highly expressed by CSCs compared with the non-CSC cell population of ATC [204,205]. Treatment with doxorubicin gradually killed the ATC-derived non-CSC population of cancer cells. This effect consequently conferred a growth advantage to CSCs, which in turn overgrew the experimental culture [204]. Inhibition of ABCG2 and/or ABCB1 unveiled that the resistance of CSCs to doxorubicin could be mainly due to the expression of these ABC transporters that were greatly up-regulated in the resistant cell subpopulation. The poor chemotherapy outcome with doxorubicin in ATC may be partially explained by the transporters of ABCG2 and ABCB1 up-regulation, which confers resistance to CSCs [204]. Thus, effective therapy for anaplastic subtype thyroid cancer will be required not only to destroy non-stem cancer cells that comprise the tumor bulk but also CSCs that represent the driving force of tumor progression.

The procedure of choice for most of DTCs is still total thyroidectomy. Total thyroidectomy has a major advantage since it can facilitate metastatic disease detection with post-surgical whole-body iodine scintigraphy (WBS), which is followed, whenever necessary, by the use of radioactive iodine (RAI) as adjuvant therapy. Lymph nodes in the neck areas are frequently involved in 20–90% of PTC cases. Microscopic metastasis is also commonly found in approximately 38–45% of cases undergoing the prophylactic central compartment dissection [206]. The sodium-iodide symporter (NIS) is an intrinsic plasma membrane protein. It can mediate active iodine transport into the thyroid gland as well as into several extrathyroidal tissues [207]. RAI therapy is based on the principle of NIS-positive cells having the ability of trapping circulating RAI and is the cornerstone of treatment for DTC residual and metastatic disease. Downregulation of the NIS gene (*SLC5A5*) leads to RAI therapy resistance [208]. The development of DTC dedifferentiation could impact functional NIS expression, thereby diminishing the efficacy of RAI therapy in dedifferentiated DTC. Genetic abnormalities, such as BRAF and RET/ PTC rearrangement, have been widely recognized as being highly responsible for the initiation, progression, and dedifferentiation of PTC, as reviewed above. These genetic abnormalities have been considered to associate with the expression decrease of iodide-handling genes in thyroid cancer, particularly the NIS gene, interfering with iodine uptake and leading to RAI therapy resistance [209]. A body of evidence has showed a strong association between the mutation of *BRAF^Val600Glu^* and the loss of RAI-avidity in PTC tumors, which may provide a sensible explanation for the RAI therapy ineffectiveness in *BRAF^Val600Glu^*-mutant PTC [210]. Moreover, RET/PTC rearrangement in thyroid cells could reduce the expression level of thyroid differentiation markers, including TSH receptor, NIS, and thyroglobulin [211,212]. Exogenous expression of RET/PTC could significantly decrease the expression of PAX8 and the activity of protein kinase A, causing diminished NIS expression [211,213]. Furthermore, IGF-1 could suppress the TSH-dependent NIS expression and lower the iodide uptake in thyroid cell lines through activation of the PI3K/AKT pathway [214]. Efforts have been made to restore the expression of NIS to achieve enhancement of RAI avidity in RAI-resistant (RAIR) DTC. Several agents, including retinoic acid (RA) [215] and PPARγ agonists [216], have been attempted to regulate the NIS gene at its transcriptional level but revealed limited clinical value in RAIR-DTC patients with the redifferentiation therapy. Selumetinib, a MEK inhibitor, was capable of increasing iodine uptake in 12 of 20 patients. Eight of the 12 patients achieved the dosimetry threshold for RAI therapy [217]. Specific kinase inhibitors targeting MAPK and PI3K signaling pathways have demonstrated promising effects in thyroid cancer treatment and shed light on the future management of RAIR-DTC patients with therapy, potentially either combining kinase inhibitors with different targets or combining kinase inhibitors with RAI.

## 6. Conclusions

The heterogeneity of thyroid cancer presents a clinical challenge, especially considering the intratumor variability. In this review, we have discussed the phenotypic heterogeneity of different subtypes of thyroid cancers. Tumor cell phenotypes are the results of the integration of genotype, environmental stimulation, and stochastic process. Genetic and epigenetic alterations during tumor initiation and progression change and diversify cellular phenotypes, posing a significant obstacle to the delineation of the mechanism and clinical management of thyroid cancers. The existence of intratumor heterogeneity is well defined in advanced thyroid cancers, and it can occur at any step of the cancer development, including early-stage tumors. With the understanding of *BRAF, RAS* mutations, and RET/PTC rearrangements in thyroid cancer, researchers still need to identify the existence of unknown genetic or epigenetic alterations within the subpopulation of CSCs. Next-generation sequencing, especially single-cell sequencing techniques, have been applied to thyroid cancer research and will establish the foundation for the development of more effective targeting therapy as these tools allow a better understanding of different clonal cancer cell fractions that bear critical genetic and/or epigenetic alterations. Novel targeted cancer therapies should be highly selective for specific genetic or epigenetic alterations; the assessment of the genetic and epigenetic patterns of thyroid cancers is therefore becoming undoubtedly necessary. Due to the cross talk and tumor escape mechanism of genetic pathways involving in thyroid cancers, therapeutic strategies using multiple pathways targeting agents are likely to have better outcomes [218]. The diverse genetic and epigenetic changes, along with the interaction between the tumor and the surrounding microenvironment, define the tumor heterogeneity. We discussed various features of the CSC subpopulation in thyroid cancers, including tumor initiation, maintenance of tumor growth, and therapeutic resistance. Efforts to identify new strategies to erode programs that enable enhanced DNA repair in CSCs among heterogeneous cancer cells remain critical to improving the durability of therapies. Taken together, the recognition of the importance of intratumor heterogeneity combined with the rapid development of single-cell technologies will allow further improvement of the understanding of thyroid cancer biology and the design of therapeutic interventions.

## Figures and Tables

**Table 1:** Variants of papillary thyroid carcinoma.

Variants	Morphological Features
Classical PTC	Cells with eosinophilic cytoplasm and enlarged nuclei cover the papillae. The tumors present with squamous metaplasia and psammoma bodies [23,24,25,26]
Papillary microcarcinoma	Tumor foci are less than 1 cm with multifocality. 11–23% of cases present with lymph node metastasis [23,24,25]
Follicular variant	A tumor possessing both typical PTC nuclear features and follicular growth patterns [23,24,25,26]
Tall cell variant	30–50% tall cells with height as two times higher than the width and basilar oriented nuclei [23,24,25,26,27]
Oncocytic variant	A distinct brown color on gross exam with follicular or papillary architecture and abundant lymphocytic stromal infiltrate [23,24,25,26]
Columnar cell variant	Pseudostratified columnar cells with supranuclear and subnuclear cytoplasmic vacuoles [23,24,25,26,27]
Diffuse sclerosing variant	Extensive squamous metaplasia, intense fibrosis, lymphoid infiltration, and psammoma bodies. Bilaterality, multifocality, extrathyroid spread, and a higher rate of lymph node metastases [23,24,25]
Solid cell variant	Solid clusters and small papillary clusters in conjunction with a clean background, sheets of tumor cells in typical features of PTC cytology with extrathyroid spread and vascular invasion [23,24,25,26]
Clear cell variant	A papillary architecture of clear cells with PTC cytological features [23,24,25]
Cribriform-morular variant	Solid and spindle cell areas within a prominent cribriform pattern tumor with squamous morules [23,24,26]
Macrofollicular variant	Macrofollicles displaying PTC cytological features with the macrofollicular patterns also appeared in metastatic lymph nodes [23,24,25,26,27]
PTC with hobnail features	The hobnail features in 30% of tumor, including diffuse sclerosing patterns as well as tall cells, very aggressive, associated with metastases [23,24,25,26,27]
PTC with fasciitis-like stroma	Tumor comprising fasciitis like or fibromatosis-like stroma [23,24,25]
Combined PTC and MTC	With mixed features of MTC and PTC; could be multicentric [24]
PTC with dedifferentiation to ATC	With areas of dedifferentiation or transformation to ATC, consisting of a mixture of pleomorphic giant cells, epithelioid cells as well as spindle cells [23,24]

PTC: papillary thyroid carcinoma; MTC: medullary thyroid carcinoma; ATC: anaplastic thyroid carcinoma.

**Table 2:** CSC isolation in thyroid carcinomas.

Method of Isolation	Mechanism of Isolation	Subject of Isolation
Cell surface marker-based cell sorting	CD44 and CD133 expression	Cancer cell lines, clinical specimens
Side population	Hoechst 33342 exclusion	Cancer cell lines
Cytoprotective enzyme-based sorting	Aldehyde dehydrogenase positivity	Cancer cell lines, clinical specimens
Cell culture selection	Serum-free and growth factor-dependent sphere formation	Clinical specimens

## Data Availability

The data that support the findings of this study are available from the corresponding author upon reasonable request.
